# Role of Mn^2+^ Doping in the Preparation of Core-Shell Structured Fe_3_O_4_@upconversion Nanoparticles and Their Applications in T_1_/T_2_-Weighted Magnetic Resonance Imaging, Upconversion Luminescent Imaging and Near-Infrared Activated Photodynamic Therapy

**DOI:** 10.3390/nano8070466

**Published:** 2018-06-26

**Authors:** Yang Luo, Wei Zhang, Zhengfang Liao, Shengnan Yang, Shengtao Yang, Xinhua Li, Fang Zuo, Jianbin Luo

**Affiliations:** College of Chemistry & Environment Protection Engineering, Southwest Minzu University, Chengdu 610041, China; polymerluoyang@163.com (Y.L.); w070812138@163.com (W.Z.); LiaoZfpolymer@163.com (Z.L.); yangsn1993@163.com (S.N.Y.); yangst@pku.edu.cn (S.T.Y.); lxh1905@126.com (X.L.)

**Keywords:** Mn^2+^, Fe_3_O_4_, upconversion, core-shell-shell, MR/UCL imaging, PDT platforms

## Abstract

Core-shell (C/S) structured upconversion coated Fe_3_O_4_ nanoparticles (NPs) are of great interest due to their potential as magnetic resonance imaging (MRI) and upconversion luminescent (UCL) imaging agents, as well as near-infrared activated photodynamic therapy (PDT) platforms. When C/S structured Fe_3_O_4_@Mn^2+^-doped NaYF_4_:Yb/Er NPs were prepared previously, well-defined C/S-NPs could not be formed without the doping of Mn^2+^ during synthesis. Here, the role of Mn^2+^ doping on the synthesis of core-shell structured magnetic-upconversion nanoparticles (MUCNPs) is investigated in detail. Core-shell-shell nanoparticles (C/S/S-MUCNPs) with Fe_3_O_4_ as the core, an inert layer of Mn^2+^-doped NaYF_4_ and an outer shell consisting of Mn^2+^-doped NaYF_4_:Yb/Er were prepared. To further develop C/S/S-MUCNPs applications in the biological field, amphiphilic poly(maleic anhydride-alt-1-octadecene) (C_18_PMH) modified with amine functionalized methoxy poly(ethylene glycol) (C_18_PMH-mPEG) was used as a capping ligand to modify the surface of C/S/S-MUCNPs to improve biocompatibility. UCL imaging, T_1_-weighted MRI ascribed to the Mn^2+^ ions and T_2_-weighted MRI ascribed to the Fe_3_O_4_ core of C/S/S-MUCNPs were then evaluated. Finally, chlorine e6 (Ce6) was loaded on the C/S/S-MUCNPs and the PDT performance of these NPs was explored. Mn^2+^ doping is an effective method to control the formation of core-shell structured MUCNPs, which would be potential candidate as multifunctional nanoprobes for future T_1_/T_2_-weighted MR/UCL imaging and PDT platforms.

## 1. Introduction

In recent years, a combination of magnetic iron oxides with upconversion to obtain magnetic-upconversion nanoparticles (MUCNPs) has received intensive attention in various biomedical fields. MUCNPs possess both useful magnetic properties and upconversion luminescent (UCL) properties. This allows them to be applied as nanoprobes for in vitro and in vivo dual-modal magnetic resonance imaging (MRI)/UCL imaging, providing high sensitivity/resolution fluorescence imaging and non-invasive and high spatial resolution MRI for real-time monitoring [1]. In addition to bioimaging, MUCNPs have also been used for drug delivery and therapy in living cells and animals. Due to their deep penetration, UCL nanoparticles conjugated with photosensitizers have become important nanocarriers for photodynamic therapy (PDT) agents [2,3].

So far, several different methods have been developed to prepare MUCNPs, such as using mesoporous silica as anintermediate layer to combine UCNPs with Fe_3_O_4_ NPs [4,5,6], polymer encapsulation [7,8], ligand crosslinking [9,10] and direct seed-nucleation [11,12,13]. This seed-growth method can be applied to construct MUCNPs by using one part as a seed onto which a second component can nucleate and grow [14,15]. The seed-growth method offers products with small sizes, homogeneous structures and tunable stoichiometry and can yield MUCNPS with core-shell (C/S) structures. On the other hand, transition metal ions, such as Mn^2+^, are often used as dopants to regulate the morphology and fluorescence intensity of UCNPs, which also play a role in T_1_-weighed MRI contrast agents.

Based on these works, we previously prepared Mn^2+^-doped C/S-MUCNPs (Fe_3_O_4_@Mn^2+^-doped NaYF_4_:Yb/Er NPs) using seed-induced growth. In this previous study, the fluorescence intensity of MUCNPs decreased due to the existence of Fe_3_O_4_, which was caused by an energy transfer process [16]. The Mn^2+^ ions also play an important role in the formation of C/S-MUCNPs; if the Mn^2+^ ions are absent, MUCNPs cannot be obtained with high quality.

**Scheme 1:**
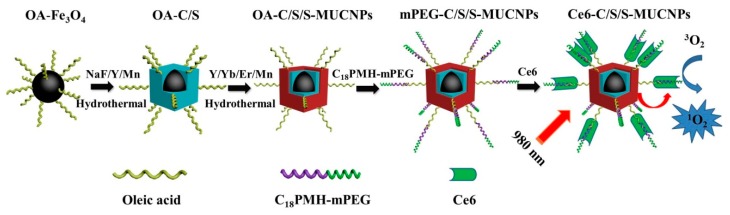
Schematic representation of the formation of Ce6-C/S/S-MUCNPs and the near-infrared (NIR) targeting photosensitizer to generate singlet oxygen species for photodynamic therapy (PDT).

In the present work, we studied the effect of Mn^2+^ doping on the formation of C/S-MUCNPs. We prepared core-shell-shell (C/S/S) nanoparticles (Fe_3_O_4_@Mn^2+^-doped NaYF_4_@Mn^2+^-doped NaYF_4_:Yb/Er NPs) with Fe_3_O_4_ as the core, an inert layer of Mn^2+^ doped NaYF_4_ and an outer shell of Mn^2+^-doped NaYF_4_:Yb/Er. We hypothesized that the inert layer of Mn^2+^-doped NaYF_4_ could shield the Fe_3_O_4_ core from the outer upconversion shell, which would decrease the quenching induced by the presence of Fe_3_O_4_. Furthermore, Mn^2+^-doped UCNPs were then used as imaging agents and drug carriers for PDT. Amphiphilic poly(maleic anhydride-alt-1-octadecene) (C_18_PMH) modified with amine functionalized methoxy poly(ethylene glycol) (C_18_PMH–mPEG), was then used to modify the C/S/S-MUCNPs in order to impart good biocompatibility. Finally, the obtained hydrophilic MUCNPs were used as contrast agents for T_1_/T_2_-weighted MRI and UCL imaging as well as near-infrared (NIR) activated PDT agents. The overall synthesis procedure is illustrated in Scheme 1.

## 2. Materials and Methods

### 2.1. Materials

All starting chemicals were obtained from commercial supplies. Ferrous sulfate heptahydrate (reagent grade, 99%) were supplied by Jinshan Chemical Technology Co., Ltd (Chengdu, China). Yttrium nitrate hexahydrate (99.99%), Ytterbium nitrate (99.99%) and Erbium trinitrate pentahydrate (99.9%) were purchased by Best Chemical Reagent Co., Ltd (Chengdu, China). Oleic acid (OA) (technical grade, 98.0%), sodium fluoride (reagent grade, 98.0%), sodium hydroxide (reagent grade, 96%), ethanol (reagent grade, 99.7%), ferric trichloride hexahydrate (reagent grade, 99.0%), manganese dichloride tetrahydrate (reagent grade, 98.0%), dimethyl sulfoxide (DMSO) were supplied by Kelong Chemical Technology Co., Ltd (Chengdu, China). Toluene (reagent grade, 99.5%), chloroform (reagent grade, 99.0%) and dichloromethane (reagent grade, 99.0%) were purchased from Ruijinte Chemical Technology Co., Ltd (Tianjin, China). Ammonia solution (reagent grade, 28%) and triethylamine (TEA) were supplied by Kelong Chemical Technology Co., Ltd. Poly(maleicanhydride-alt-1-octadecene) (C_18_PMH) were supplied by Sigma-Aldrich Co., LLC. (Shanghai, China). Methoxy poly(ethylene glycol) amine (mPEG-NH_2_) were supplied by Aladdin company (Shanghai, China). 1-ethyl-3-(3-(dimethylamino)propyl) carbodiimide hydrochloride (EDC·HCl) and 1,3-Diphenylisobenzofuran (DPBF) were obtained from Best Chemical Reagent Co., Ltd. Chlorin e6 were supplied by Frontier Scientific (Logan, UT, USA).

### 2.2. Synthesis of OA Coated Fe_3_O_4_@NaYF_4_:xMn (x = 0, 30 mol %) NPs

First, the hydrophobic OA coated Fe_3_O_4_ NPs were prepared according to our previously reported procedure and then dispersed in toluene for use in the next step [17]. The as-prepared OA-Fe_3_O_4_ NPs were used as seeds and covered with shell according to our previously reported method [18]. In the case of Fe_3_O_4_@NaYF_4_:xMn (x = 30 mol %) NPs, as an example, 1.42 mL of 0.5 M MnCl_2_, 3.31 mL of 0.5 M Y(NO_3_)_3_ water solution were added to a mixture of NaOH (1.0 g), 5.0 mL toluene dispersion of OA-Fe_3_O_4_ (12 mg/mL), OA (16.72 mL) and ethanol (33.55 mL) under stirring. Then 10 mL of deionized water containing 0.68 g NaF was drop wisely added into the mixture. After vigorous stirring for 30 min, the colloidal solution was transferred into a 100 mL Teflon-lined autoclave (Guo wei technology limited, Chengdu, China), which was sealed and heated at 200 °C for 8 h and then the mixture was cooled down to room temperature. The final product was collected by magnetic-separation and then washed with ethanol and deionized water for several times. Finally, the obtained OA-coated Fe_3_O_4_@NaYF_4_:xMn (x = 30 mol %) NPs (G1-NaYF_4_-Mn^2+^ MUCNPs) were dispersed in toluene.

### 2.3. Synthesis of OA Coated Fe_3_O_4_@NaYF_4_:18%Yb/2%Er/xMn(x = 0, 30 mol %) NPs

The OA coated Fe_3_O_4_@NaYF_4_:18%Yb/2%Er/xMn (x = 0, 30 mol %) NPs were also prepared according to our previously reported method [18]. In the procedure, the obtained OA coated Fe_3_O_4_@NaYF_4_:18%Yb/2%Er/xMn (x = 30 mol %) NPs (G2-NaYF_4_-Mn^2+^ MUCNPs) and OA coated Fe_3_O_4_@NaYF_4_:18%Yb/2%Er/xMn (x = 0 mol %) NPs (G2-NaYF_4_ MUCNPs) were dispersed in toluene.

### 2.4. Synthesis of OA Coated Fe_3_O_4_@NaYF_4_:xMn@NaYF_4_:18%Yb/2%Er/xMn(x = 30 mol %) Core/Inert Shell/Active Shell Structured MUCNPs

To form the second shell, the as-prepared G1-NaYF_4_-Mn^2+^ MUCNPs were used as the nucleation seeds for growth of the active capping layer. The G1-NaYF_4_-Mn^2+^ MUCNPs were used as seeds and covered with an active shell using a facile hydrothermal method according to our previously reported method [18]. The final product was collected by magnetic-separation and then washed with ethanol and deionized water for several times. Finally, the obtained OA-Fe_3_O_4_@NaYF_4_/xMn@NaYF_4_:18%Yb/2%Er/xMn (x = 30 mol %) NPs (OA-C/S/S-MUCNPs) were dispersed in chloroform.

### 2.5. Synthesis of mPEG-Functionalized Fe_3_O_4_@NaYF_4_:xMn@NaYF_4_:18%Yb/2%Er/xMn(x = 30 mol %) Core/Inert Shell/Active Shell Structured MUCNPs

The synthesis of mPEG-Fe_3_O_4_@NaYF_4_:xMn@NaYF_4_:18%Yb/2%Er/xMn (x = 30 mol %) NPs (mPEG-C/S/S-MUCNPs) was carried out according to a published method [19,20,21]. At first, C_18_PMH-mPEG was synthesized according to a published protocol [22,23]. C_18_PMH-mPEG (15 mg) and OA coated Fe_3_O_4_@NaYF_4_:xMn@NaYF_4_:18%Yb/2%Er/xMn (x = 30 mol %) NPs (15 mg) were dispersed separately in chloroform (10 mL) and then mixed together to obtain a homogeneous phase. The mixture was stirred for 2 h at room temperature. After magnetic separation, the black solid was redispersed in 10 mL of water and the large aggregates were removed using a 0.22-μm drainage membrane filter and mPEG-C/S/S-MUCNPs were stored at 4 °C.

### 2.6. Synthesis of mPEG-Fe_3_O_4_@NaYF_4_:xMn@NaYF_4_:18%Yb/2%Er/xMn (x = 30 mol %) Core/Inert Shell/Active Shell Structured MUCNPs loaded with Ce6

According to a previously reported method [24], chlorin e6 (Ce6) molecules were loaded onto mPEG-C/S/S-MUCNPs via hydrophobic interactions. Briefly, 5 mg Ce6 was dissolved in 5 mL DMSO as the stock solution. Ce6 with the desired concentration (1 mg/mL) was added into mPEG-C/S/S-MUCNPs (0.5 mg/mL) aqueous solution. The mixture was placed at room temperature with continuous stirring, in the dark, overnight. The samples were removed after magnetic separation and washed 3 times with deionized water. The acquired mPEG-Fe_3_O_4_@NaYF_4_:xMn@NaYF_4_:18%Yb/ 2%Er/xMn (x = 30 mol %) NPs loaded with Ce6 (Ce6-C/S/S-MUCNPs) complex was redispersed in water by sonication and stored at 4 °C in the dark.

### 2.7. Cytotoxicity Assay

The effect of mPEG-C/S/S-MUCNPs on cell viability was determined using MTT assay [25,26]. HeLa cells were seeded into 96-well plates at a density of 1 × 10^4^/mL (200 μL of fresh medium per well) and cultured at 37 °C and 5% CO_2_. The cell culture medium in each well was then replaced by 200 μL of cell growth medium containing different concentrations of mPEG-C/S/S-MUCNPs (31.25, 62.50, 125, 250, 500 µg/mL). After incubation for 24 h, 200 μL of MTT (0.5 mg/mL in a phosphate-buffered saline solution) was added to each well for another 4 h at 37 °C. The growth medium was removed gently using suction, 300 μL of DMSO was added to each well as a solubilizing agent and the microplate was left at room temperature for 2 h.

### 2.8. In Vivo UCL Imaging

Firstly, 50 μL of mPEG-C/S/S-MUCNPs (500 μg/mL) were injected intradermally into the subcutaneous tissue of nude mice. The system includes two external adjustable CW 980 nm lasers (0*–*5 W) (Shanghai Connet Fiber Optics Co., Shanghai, China) sources and an Andor DU897 EMCCD as the signal collector. Images of luminescent signals were processed with the Kodak Molecular Imaging software (Rochester, NY, USA).

### 2.9. In Vitro T_1_/T_2_-Weighted MRI

Initially, 1 mL of the mPEG-C/S/S-MUCNPs solution at different Mn^2+^ concentrations or Fe concentrations were prepared before MRI. Both T_1_-weighted and T_2_-weighted magnetic resonance (MR) images were acquired using a 7.0 T MRI scanner (Bruker BioSpec 70/20 USR, Karlsruhe, Germany). The parameters refer to our previous method [27].

### 2.10. Characterization

The morphology and size of the as-prepared MUCNPs were characterized by a Tecnai F20 Transmission Electron Microscopy (TEM) (Beijing, China) with an attached energy dispeisive X-ray (EDX) spectroscopy stytem. Powder X-ray diffraction measurements were performed on a XD-6 diffractometer (Persee, Beijing, China) at a scanning rate of 8° min^−1^ in the 2θ range from 10° to 90° (Cu Kαline λ = 0.154 nm). The magnetic properties were assessedon a Vibrating Sample Magnetometer (VSM, HH-15, Nanjing, China) at 298 K under an applied magnetic field. The UCL spectra were performed by Hitachi F-4600 fluorescent spectrometer (Shanghai, China) using 980 nm diode laser. Fourier Transform Infrared Spectra (FTIR) were on Spectrum One (Chengdu, China) with KBr pellet in the spectral range of 4000 to 500 cm^−1^. SDTA 851C Thermogravimetric Analyzer (TGA) (Chengdu, China) was used to analysis of upconversion nanoparticles. An inductively coupled plasma mass spectrometry (ICP-MS) system (Shimadzu Corp., Kyoto, Japan) was used to analyze the element concentration of the MUCNPs.

## 3. Results

### 3.1. Influence of Mn^2+^ Doping on the Formation of Core-Shell Structured MUCNPs

In our previous work, the C/S-MUCNPs were prepared using a seeded-growth approach [18]. The obtained product precipitated in the absence of Mn^2+^ doping, indicating that the doping of Mn^2+^ plays an important role in the formation of C/S-MUCNPs with high-quality.

**Figure 1:**
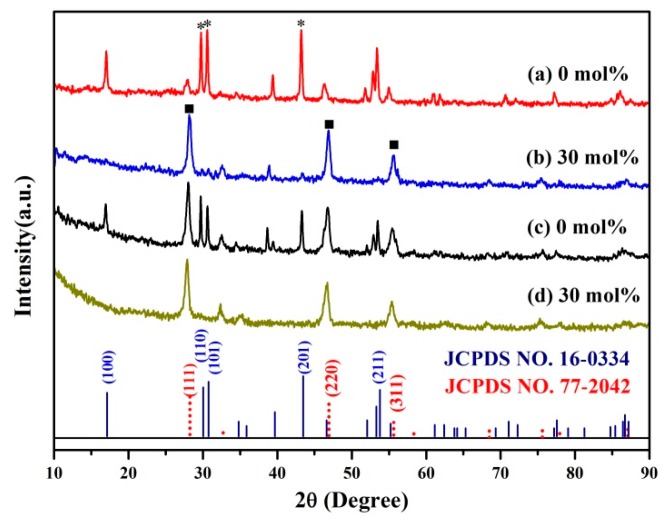
X-ray diffraction patterns of G1-NaYF_4_ (**a**) and G1-NaYF_4_-Mn^2+^ (**b**); G2-NaYF_4_ (**c**) and G2-NaYF_4_-Mn^2+^ (**d**). The diffraction peaks of the cubic phase are marked with ■ and peaks related to the hexagonal phase are marked with *.

**Figure 2:**
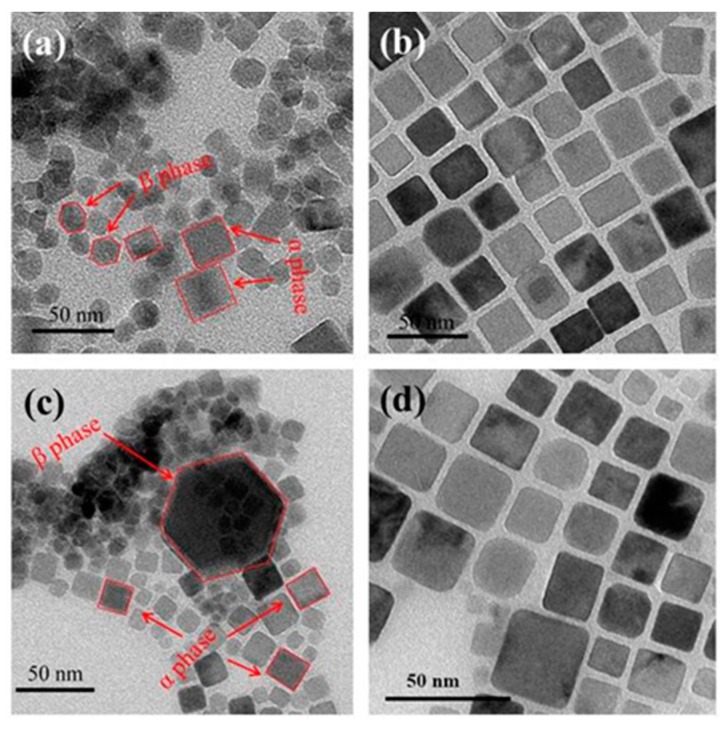
Transmission electron microscopy (TEM) images of G1-NaYF_4_ (**a**) and G1-NaYF_4_-Mn^2+^ (**b**); G2-NaYF_4_ (**c**) and G2-NaYF_4_-Mn^2+^ (**d**).

To explore the effect of Mn^2+^-doping on the synthesis of C/S-MUCNPs, two groups of experiments were designed here; one group was synthesized in the presence of Y^3+^ with (G1-NaYF_4_-Mn^2+^) or without Mn^2+^ doping (G1-NaYF_4_) and the other group was synthesized in the presence of Y^3+^, Yb^3+^ and Er^3+^ with (G2-NaYF_4_-Mn^2+^) or without Mn^2+^ doping (G2-NaYF_4_). Figure 1 shows X-ray diffraction patterns (XRD) of the as-prepared products. Without Mn^2+^doping, the product possesses a cubic phase (JCPDS No. 77-2042, marked with ■) and a hexagonal phase (JCPDS No. 16-0334, marked with *) as shown in Figure 1a,c. Furthermore, the phase transformation from the coexistence of α and β-phases to pure α-phase was completed when 30 mol % Mn^2+^ doping was used, as shown in Figure 1b,d. The reflection peak intensity of Fe_3_O_4_ contained in these products was relatively weak, likely because the magnetic materials in the composites occurred in a low concentration or had a low level of crystallization. The size and morphology of the as-prepared products were furthermore characterized by transmission electron microscopy (TEM). Figure 2a,c show TEM images of the obtained product prepared in the absence of Mn^2+^, where the samples were aggregated and formed a mixture of NaYF_4_ with α and β-phases and isolated Fe_3_O_4_ NPs. Figure 2b,d show TEM images of the samples prepared with 30 mol % Mn^2+^ doping, showing that high quality and uniform NPs with pure cubic phase structures were obtained.

The TEM and XRD results indicated that if the upconversion part contained β-phases, the obtained product was deposited and low-quality products would be obtained. On the other hand, if the upconversion part featured only cubic phases, high-quality C/S-MUCNPs were obtained. Blower and co-workers demonstrated that C/S structured Fe_3_O_4_@NaYF_4_ (Yb/Er), could be formed by growing the (011) plane of cubic NaYF_4_ on the (111) plane of Fe_3_O_4_ with a rotation angle of 30° [13]. Meanwhile, Yan and co-workers showed that hexagonal NaYF_4_ could not be directly formed on the surface of Fe_3_O_4_ due to the lattice mismatch between Fe_3_O_4_ and hexagonal NaYF_4_ [28].

According to these previous works and the findings of this study, Mn^2+^ induced hexagonal-to-cubic phase transformations and the formed cubic phase grew on the surface of Fe_3_O_4_ to obtain MUCNPs with a C/S structured.

To determine why the precipitation only takes place in the absence of Mn^2+^, additional experiments were conducted in which only NaF was introduced into this system in the presence of oleic acid coated Fe_3_O_4_ (OA-Fe_3_O_4_) NPs, precipitating the obtained product. X-ray photoelectron spectra (XPS) were then used to examine the composition of the precipitated product. As shown in Figure S1, a peak at 685.42 eV assigned to F-1s was found in the XPS spectra, indicating that F^−^ ions were absorbed onto the surface of Fe_3_O_4_ NPs. According to these results, the F^-^ ions likely induce the desorption of OA bound onto the Fe_3_O_4_, in the presence of Mn^2+^, causing nucleation and growth to occur on the surface of Fe_3_O_4_, followed by OA molecules being absorbed on the formed upconversion shell to stabilize the C/S-MUCNPs. Without Mn^2+^, the rare earth ions would nucleate and grow into isolated UCNPs with lattice mismatch between Fe_3_O_4_ and hexagonal NaYF_4_ and the Fe_3_O_4_ NPs would aggregate and deposit due to the loss of stabilization provided by OA.

According to these results, the mechanism of the C/S-MUCNPs synthesis could be described as follows. The addition of NaF induced the desorption of OA molecules from the surface of Fe_3_O_4_ NPs and then Mn^2+^ induced the nucleation and growth of the upconversion shell on the Fe_3_O_4_ NPs, which resulted in the formation of C/S-MUCNPs stabilized by OA molecules. The proposed synthesis mechanism is illustrated in Scheme S1.

### 3.2. Preparation of Core/Inert Shell/Active Shell Structured MUCNPs

**Figure 3:**
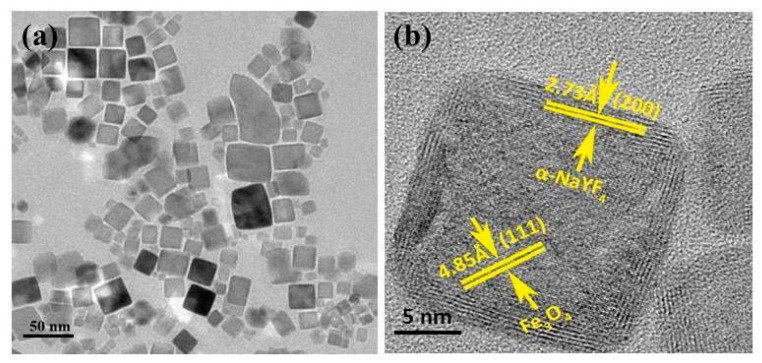
TEM image (**a**) and HRTEM image (**b**) of C/S/S-MUCNPs.

As discussed previously, an inert layer between Fe_3_O_4_ NPs and luminescent shells in C/S structured luminescent and magnetic NPs could decrease the quenching of luminescence by the Fe_3_O_4_ core. Core/inert shell/active shell structured MUCNPs were designed in this study, where Fe_3_O_4_ NPs were coated by an inert shell of Mn^2+^-doped NaYF_4_, onto which the active UCL shell made of Mn^2+^ doped NaYF_4_:Yb/Er was formed. In the following description, C/S/S-MUCNPs refers to the core/inert shell/active shell structured MUCNPs. The composition and structure of the C/S/S-MUCNPs were characterized by XRD and TEM, respectively. Figure S2 shows the XRD spectra of the C/S/S-MUCNPs, where the diffraction peaks were indexed to cubic-phase NaYF_4_ (JCPDS card No. 77-2042). Figure 3a shows TEM images of the C/S/S-MUCNPs, compared with that of the C/S structured Fe_3_O_4_@Mn^2+^-doped NaYF_4_ NPs. The size was increased, indicating the formation of another shell on the inert shell of Mn^2+^-doped NaYF_4_. The fringe distances, measured using high resolution TEM (HRTEM) (Figure 3b), were 4.85 Å and 2.73 Å for the two types of grains, which matched well with the known lattice constants for Fe_3_O_4_ and cubic NaYF_4_ phases, respectively. This confirms the successful coating of Fe_3_O_4_ with a cubic upconversion shell.

**Figure 4:**
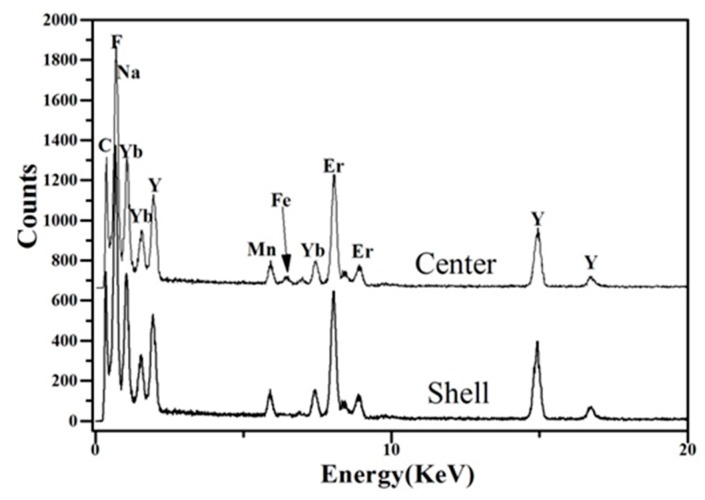
EDX analysis of the central and shell region of C/S/S-MUCNPs.

To further confirm the formation of the composite structure, energy dispersive X-ray (EDX) was conducted, where the results are shown in Figure 4. The EDX result clearly indicated that Fe, F, Na, Y, Yb, Er and Mn were detected in the central region, while no Fe signal was obtained from the shell part. The data indicated that C/S/S-MUCNPs magnetic-fluorescent nanocomposites were successfully prepared. Other signals came from the TEM grid.

**Figure 5:**
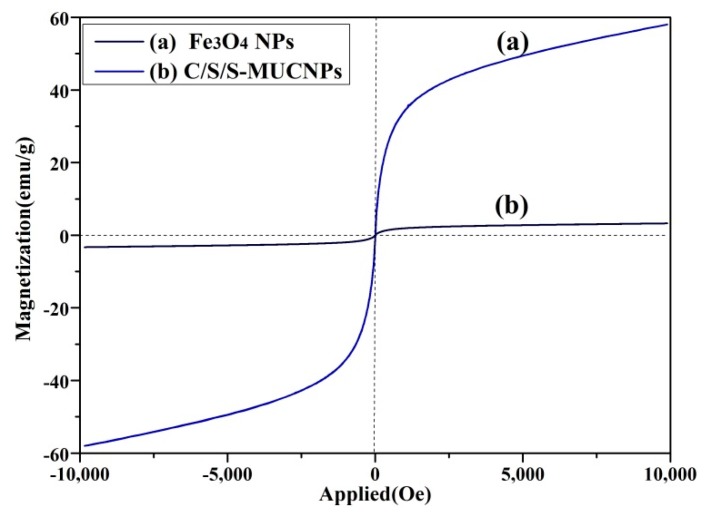
Room-temperature magnetic hysteresis loop for Fe_3_O_4_ NPs (**a**) and C/S/S-MUCNPs (**b**).

The magnetic properties of the C/S/S-MUCNPs and original Fe_3_O_4_ NPs were then characterized with a VSM magnetometer. After the formation of the C/S/S-MUCNPs, the saturation magnetization was significantly decreased to 3.85 emu·g^−1^ (Figure 5). This phenomenon was mainly caused by the presence of a high proportion of upconversion in the composite NPs. Fortunately, both the C/S/S-MUCNPs and the original Fe_3_O_4_ nanoparticles display superparamagnetic behavior (zero coercivity and resonance), which is crucial for use biomedicine and biotechnology applications [29]. In the future, we plan to carry out a detailed study to improve the saturation magnetization of MUCNPs.

**Figure 6:**
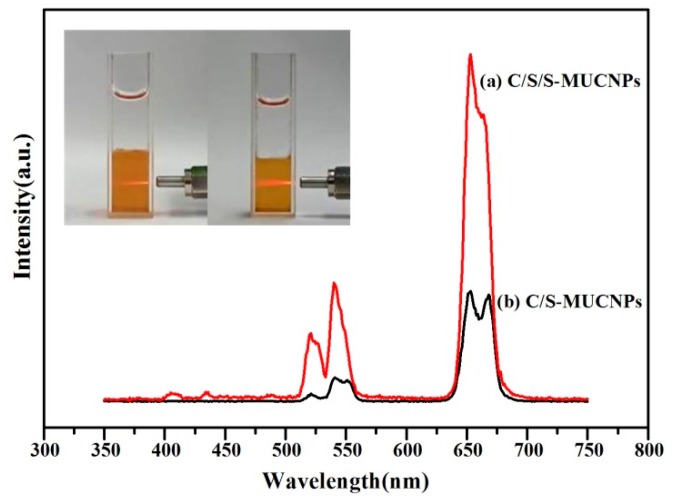
Upconversion emission spectra of C/S/S-MUCNPs (**a**) and C/S-MUCNPs (**b**). The inset shows photographic images of the chloroform solution of MUCNPs under 980 nm irradiation, C/S-MUCNPs (left) and C/S/S-MUCNPs (right).

The optical properties of the C/S/S-MUCNPs were also investigated. A higher UCL emission intensity was clearly observed for C/S/S-MUCNPs compared to the C/S-MUCNPs (Figure 6), due to the existence of the inert shell which prevented the fluorescence from being quenched by Fe_3_O_4_ [30]. The UCL spectra of C/S/S-MUCNPs in chloroform (Figure 6) exhibited two peaks at 550 nm (green emission peak) and 660 nm (red emission peak), where the red to green intensity ratio (IR/IG) was 3.13. The intense red light (650–670 nm) luminescence was ascribed to the presence of Mn^2+^ [31,32].

The C/S/S-MUCNPs described above, which were inevitably covered with OA, were converted to a water-soluble form by coating them with amphiphilic polymer (poly(maleic anhydride-alt-1-octadecene) modified with methoxy poly(ethylene glycol) amine (C_18_PMH-mPEG)), as shown in Figure S3. The appearance of characteristic peaks associated with the PEG chain at 1107 and 952 cm^−1^ in the Fourier transform infrared (FTIR) spectra of PEGylated NPs (Figure S3) and a mass loss of up to 30.7% starting from >200 °C on thermogravimetric curves (Figure S4), confirmed the coating of C_18_PMH-mPEG. As shown in Figure S2, the XRD spectra indicated that the C_18_PMH-mPEG coated C/S/S-MUCNPs (mPEG-C/S/S-MUCNPs) maintained a cubic phase structure (JCPDS Card No. 77-2042). In addition, the mPEG-C/S/S-MUCNPs were separated from each other without observable aggregation, suggesting that they were effectively stabilized in the aqueous solution (Figure S5).

### 3.3. In Vitro UCL Imaging and In Vitro T_1_/T_2_-Weighted MRI Imaging

**Figure 7:**
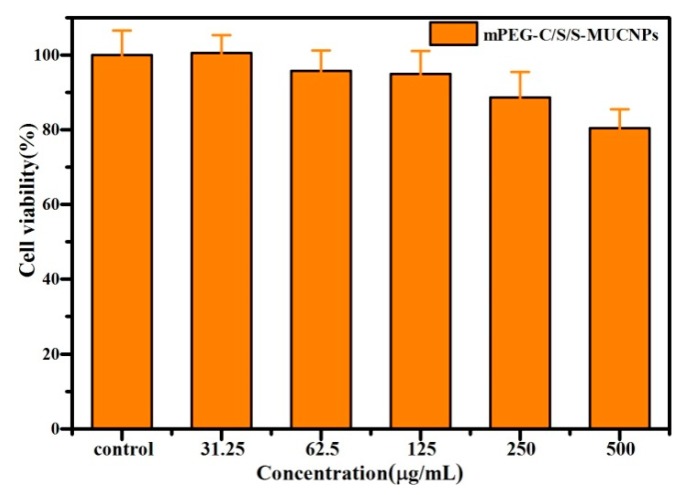
In vitro cytotoxicity of mPEG-C/S/S-MUCNPs against HeLa cells at different concentrations after 24 h incubation.

Prior to in vitro and in vivo biological applications, the cytotoxicity of the obtained mPEG-C/S/S-MUCNPs was measured using an MTT assay, an approach widely used to measure mitochondria activity to quantify cell growth or cell death [33]. The viability of the untreated cells was assumed to be 100%. Figure 7 shows the cell viability after 24 h of incubation with the mPEG-C/S/S-MUCNPs at different concentrations from 31.25 to 500 μg·mL^−1^. After 24 h of incubation at a concentration of 500 μg·mL^−1^, the cells viability was approximately 81%. These results indicate that the prepared mPEG-C/S/S-MUCNPs exhibit low toxicity.

**Figure 8:**
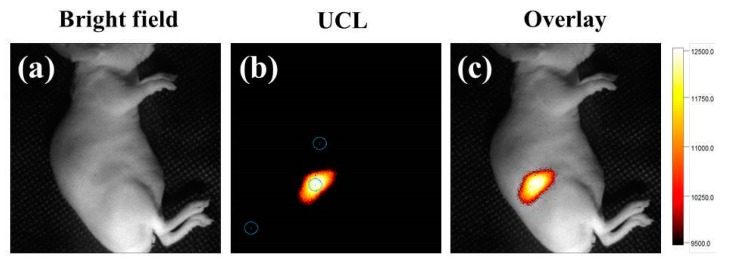
In vivo upconversion luminescence (UCL) imaging. Bright field image (**a**); UCL image after injection with mPEG-C/S/S-MUCNPs (0.5 mg/mL, 50 μL) under irradiation at 980 nm (power density of 300 mW/cm^2^, λ emission = 660 ± 12 nm) (**b**); merged image of bright field image and UCL signal (**c**).

UCL imaging is a non-invasive and sensitive method to visualize morphological details of biological samples [34,35,36]. To verify the capacity of the mPEG-C/S/S-MUCNPs for UCL imaging, we injected mPEG-C/S/S-MUCNPs into the thighs of mice. UCL imaging was achieved from the injected sites using an EMCCD camera, where excitation was provided by a CW infrared laser at 980 nm and UCL signals were collected at 660 ± 12 nm. The strong NIR UCL signal was detected at the mice thigh by the in vivo Maestro (CRI) imaging system upon excitation at 980 nm (Figure 8b). The overlay image (Figure 8c) with high signal-to-noise ratio (SNR = 18.26) further confirmed that the mPEG-C/S/S-MUCNPs were an excellent probe for in vivo NIR UCL imaging.

**Figure 9:**
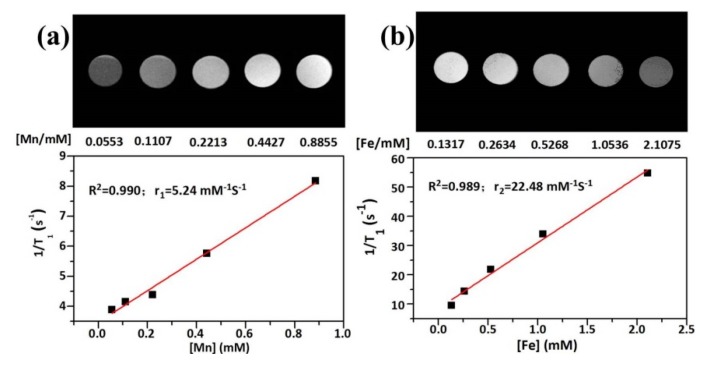
In vitro T_1_-weighted image of mPEG-C/S/S-MUCNPs and a relaxivity plot of r_1_ vs. Mn contents (**a**). In vitro T_2_-weighted image of mPEG-C/S/S-MUCNPs and a relaxivity plot of r_2_ vs. Fe contents (**b**).

The elemental composition of mPEG-C/S/S-MUCNPs was analyzed by ICP-MS, where the Fe/Mn molar ratio was estimated to be 2.38:1. In order to evaluate the ability of mPEG-C/S/S-MUCNPs as a T_1_–T_2_ dual modal MRI agent, the transverse relaxivity (r_2_) and longitudinal relaxivity (r_1_) were determined by plotting the inverse relaxation time against the Fe and Mn concentration, respectively. The r_1_ was 5.24 mM^−1^ s^−1^ which was calculated from the curve of 1/T_1_ versus the Mn^2+^ concentration (Figure 9a), Meanwhile, the r_2_ was calculated to be 22.48 mM^−1^ s^−1^ (Figure 9b). These results suggested that the NPs could be used for both T_1_- and T_2_-weighted MR imaging. In future studies, we will investigate the effect of Mn and Fe contents and the interaction between Mn-doped NaYF_4_ and Fe_3_O_4_ on the r_2_/r_1_ ratio to make these MUCNPs viable for use in MRI imaging.

### 3.4. Evaluation of PDT Performance of C/S/S-MUCNPs Loaded with Ce6

**Figure 10:**
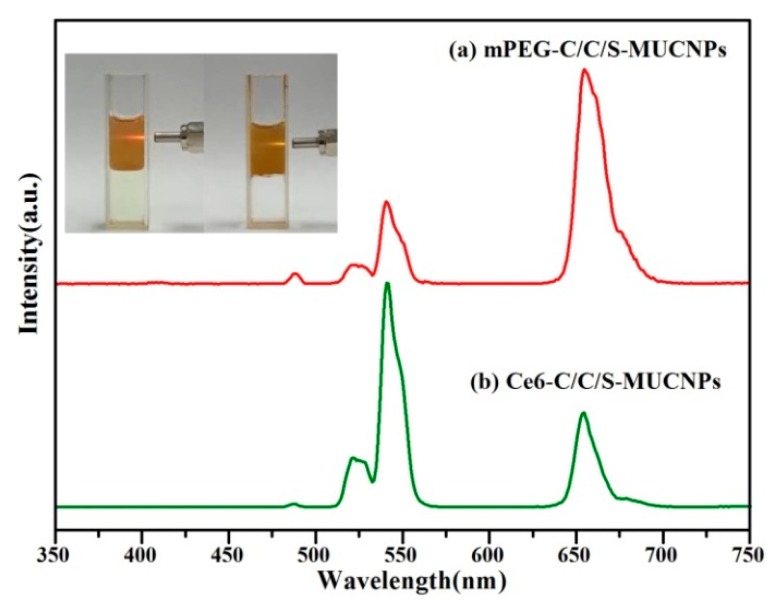
UCL spectra of mPEG-C/S/S-MUCNPs (**a**) and Ce6-C/S/S-MUCNPs (**b**) under the excitation of a 980 nm diode laser.

To demonstrate the feasibility of using mPEG-C/S/S-MUCNPs loaded with Ce6 (Ce6-C/S/S-MUCNPs) in PDT, Ce6 was conjugated to C/S/S-MUCNPs, as the black emission from the MUCNPs matched well with the absorption peak of Ce6 (Figure S6). To analyze the energy transfer between MUCNPs and Ce6, we measured the UC emission spectra of MUCNPs-Ce6 complexes using 980 nm excitation (Figure 10). The intensity ratio of the green emission peak (IG) to the red emission peak (IR) was about 2.2:1 for Ce6-C/S/S-MUCNPs. However, the IG:IR was only 1:2.8 for mPEG-C/S/S-MUCNPs due to resonance energy transfer from the MUCNPs to the nearby Ce6 molecules, which had an absorption peak at exactly 660 nm [37,38].

**Figure 11:**
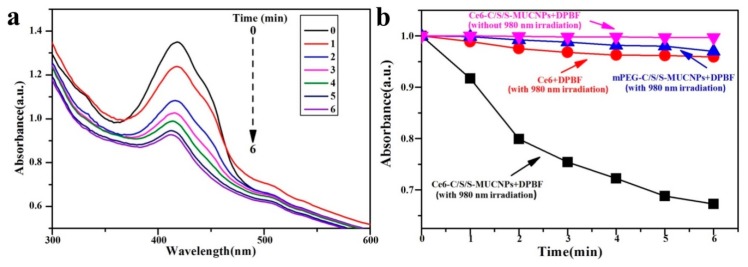
Absorption spectra of 1,3-Diphenylisobenzofuran (DPBF) under NIR irradiation in aqueous dispersion of Ce6-C/S/S MUCNPs (100 μg·mL^−1^) (**a**); Consumption of DPBF over time (black); others were control experiments without UCNPs or NIR, respectively (**b**).

Singlet oxygen is thought to be the major cytotoxic species that causes cell death through the so-called type II mechanism [39,40]. Herein, a singlet-oxygen chemical probe, 1,4-diphenyl-2,3-benzofuran (DPBF), was chosen to evaluate the ^1^O_2_ generation capability of Ce6-C/S/S-MUCNPs, owing to the fact that DPBF can react irreversibly with generated ^1^O_2_, leading to a decreased intensity of the DPBF absorption (around 410 nm) [41,42]. The changes in the absorption spectra of DPBF in the presence of Ce6-C/S/S-MUCNPs after different irradiation times are shown in Figure 11a. Control tests were carried out to confirm that the decrease in the absorption of DPBF was induced by singlet oxygen (Figure 11b). In the presence of Ce6-C/S/S-MUCNPs, the DPBF absorption at 410 nm dramatically decreased under 980 nm irradiation (Figure 11a,b), thereby suggesting that these NPs were highly efficient in the generation of reactive ^1^O_2_. In contrast, there were no obvious decreases in DPBF absorbance for solutions containing mPEG-C/S/S-MUCNPs or free Ce6 (Figure 11b). The effective ^1^O_2_-generating capability of Ce6-C/S/S-MUCNPs under NIR radiation makes them viable for NIR-induced PDT applications.

**Figure 12:**
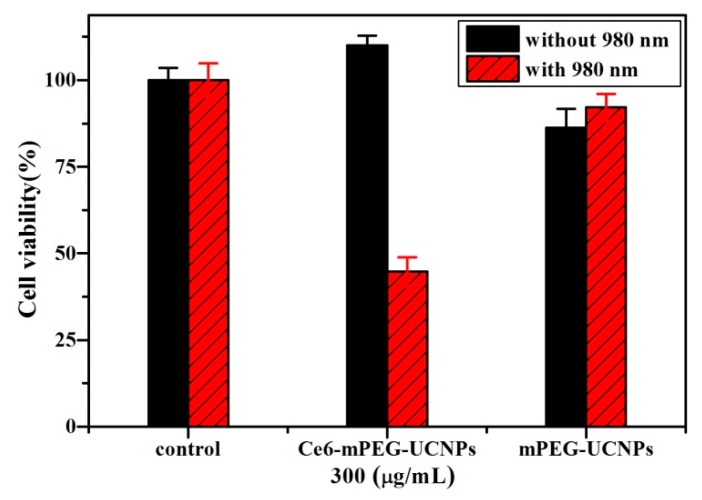
Viability of HeLa cells treated with mPEG- and Ce6-C/S/S MUCNPs at 300 μg/mL with and without (0.3 W/cm^2^) 980 nm irradiation.

The PDT efficiency of Ce6-C/S/S-MUCNPs was preliminarily evaluated on Hela cancer cells using the standard MTT assay. As shown in Figure 12, no significant cytotoxicity was observed after treatment with Ce6-C/S/S-MUCNPs in the absence of irradiation. In contrast, the cell viability of Ce6-C/S/S-MUCNPs (300 μg/mL) decreased to approximately 44% after a 10-min laser irradiation (0.3 W/cm^2^). This demonstrates that these NPs can exert severe toxicity to HeLa cells upon 980 nm laser irradiation. No reduction in cell viability was noticed for cells incubated with bare NPs in the presence of NIR irradiation (Figure 12), indicating that bare NPs with irradiation did not produce cancer cell-killing singlet oxygen. These results clearly demonstrate the feasibility of Ce6-C/S/S-MUCNPs as PDT agents.

## 4. Conclusions

In summary, Mn^2+^ doping was confirmed to be crucial for the formation of core-shell structured MUCNPs, as the doping of Mn^2+^ could induce hexagonal-to-cubic phase transformations, where only the formed cubic phase can grow on the surface of Fe_3_O_4_ NPs. Core/inert shell/active shell structured MUCNPs were then prepared, where Fe_3_O_4_ NPs were coated by the inert shell of Mn^2+^-doped NaYF_4_, on which an active UCL shell made of Mn^2+^-doped NaYF_4_:Yb/Er was formed. The inert shell prevented the UCL from being quenched by Fe_3_O_4_. Furthermore, water-dispersible C/S/S-MUCNPs were obtained by coating with an amphiphilic polymer (C_18_PMH–mPEG), which exhibited good biocompatibility. Moreover, the prepared C_18_PMH–mPEG coated C/S/S-MUCNPs could be used for in vivo UCL imaging with a signal-to-noise ratio of 18.26. The C/S/S-MUCNPs were also used as dual-mode T_1_/T_2_-weighted MRI agent because of the co-existence of Fe_3_O_4_ and Mn^2+^ in the NPs, with an r_1_ value of 5.24 mM^−1^ s^−1^ and r_2_ value of 22.48 mM^−1^ s^−1^. On the other hand, the main UCL band at 654–674 nm was matched with the excitation wavelength of the Ce6 photosensitizer, by which singlet oxygen species were generated to kill cancer cells. Under irradiation by 980 nm laser for 10 min, the cell death of HeLa cells incubated with Ce6-C/S/S-MUCNPs (300 μg·mL^−1^) could reach approximately 56%. Therefore, the as-prepared C/S/S-MUCNPs are thought to be good multifunctional agents for T_1_/T_2_-weighted MR/UCL imaging and PDT platforms for cancer treatment.

## Supplementary Materials

The following are available online at http://www.mdpi.com/2079-4991/8/7/466/s1, Scheme S1: Proposed synthetic mechanism for the formation of Fe_3_O_4_@NaYF_4_ NPs, Figure S1: XPS spectra of Fe_3_O_4_ NPs treated by hydrothermal process in the presence of NaF, Figure S2: X-ray diffraction patterns of OA-C/S/S-MUCNPs (a) and mPEG-C/S/S-MUCNPs (b), Figure S3: FTIR spectra of OA-C/S/S-MUCNPs (a) and mPEG-C/S/S-MUCNPs (b), Figure S4: TGA curves of OA-C/S/S-MUCNPs (a) and mPEG-C/S/S-MUCNPs (b), Figure S5: TEM images of OA-C/S/S-MUCNPs (a) and mPEG-C/S/S-MUCNPs (b), Figure S6: UV-Vis absorbance spectrum of Ce6-C/S/S-MUCNPs (a), Ce6 (b), and mPEG-C/S/S-MUCNPs (c).

## References

[1] Zhang L., Wang Y.S., Yang Y., Zhang F., Dong W.F., Zhou S.Y., Pei W.H., Chen H.D., Sun H.B. (2012). Magnetic/upconversion luminescent mesoparticles of Fe_3_O_4_@LaF_3_:Yb^3+^, Er^3+^ for dual-modal bioimaging. Chem. Commun..

[2] Xu F.L., Liu M.X., Li X., Xiong Z.J., Cao X.Y., Shi X.Y., Guo R. (2018). Loading of indocyanine green within polydopamine-coated laponite nanodisks for targeted cancer photothermal and photodynamic therapy. Nanomaterials.

[3] Nancy L.O., Rachel L.M., Irina B. (2002). The role of apoptosis in response to photodynamic therapy: What, where, why and how. Photochem. Photobiol. Sci..

[4] Darbandi M., Nann T. (2006). One-pot synthesis of YF_3_@silica core/shell nanoparticles. Chem. Commun..

[5] Gai S.L., Yang P.P., Li C.X., Wang W.X., Dai Y.L., Niu N., Lin J. (2010). Synthesis of magnetic, up-conversion luminescent and mesoporous core-shell-structured nanocomposites as drug carriers. Adv. Funct. Mater..

[6] Ansari A.A., Yadav R., Rai S.B. (2016). Enhanced luminescence efficiency of aqueous dispersible NaYF_4_:Yb/Er nanoparticles and the effect of surface coating. RSC Adv..

[7] Cheung E.N.M., Alvares R.D.A., Oakden W., Chaudhary R., Hill M.L., Pichaandi J.Y., Mo G.C.H., Yip C., Macdonald P.M., Stanisz G.J. (2010). Polymer-stabilized lanthanide fluoride nanoparticle aggregates as contrast agents for magnetic resonance imaging and computed tomography. Chem. Mater..

[8] Jana N.R., Patra P.K., Saha A., Basiruddin S.K., Pradhan N. (2009). Imidazole based biocompatible polymer coating in deriving <25 nm functional nanoparticle probe for cellular imaging and detection. J. Phys. Chem. C.

[9] Jiang S., Win K.Y., Liu S.H., Teng C.P., Zheng Y.G., Han M.Y. (2013). Surface-functionalized nanoparticles for biosensing and imaging-guided therapeutics. Nanoscale.

[10] Feng W., Han C.M., Li F.Y. (2013). Upconversion-nanophosphor-based functional nanocomposites. Adv. Mater..

[11] Huang Z.Y., Gao H.P., Mao Y.L. (2016). Understanding the effect of Mn^2+^ on Yb^3+^/Er^3+^ upconversion and obtaining a maximum upconversion fluorescence enhancement in inert-core/active-shell/inert-shell structures. RSC Adv..

[12] Zeng L.Y., Xiang L.C., Ren W.Z., Zheng J.J., Li T.H., Chen B., Zhang J.C., Mao C.W., Li A.G., Wu A.G. (2013). Multifunctional photosensitizer-conjugated core–shell Fe_3_O_4_@NaYF_4_:Yb/Er nanocomplexes and their applications in T_2_-weighted magnetic resonance/upconversion luminescence imaging and photodynamic therapy of cancer cells. RSC Adv..

[13] Cui X.J., Mathe D., Kovács N., Horváth I., Jauregui-Osoro M., Rosales R.T.M., Mullen G.E.D., Wong W., Yan Y., Krüger D. (2016). Synthesis, characterization and application of core–shell Co_0.16_Fe_2.84_O_4_@NaYF_4_(Yb, Er) and Fe_3_O_4_@NaYF_4_(Yb, Tm) nanoparticle as trimodal (MRI, PET/SPECT and Optical)imaging agents. Bioconj. Chem..

[14] Gu H.W., Zheng R.K., Zhang X.X., Xu B. (2004). Facile one-pot synthesis of bifunctional heterodimers of nanoparticles: A conjugate of quantum dot and magnetic nanoparticles. J. Am. Chem. Soc..

[15] Kwon K.W., Shim M. (2005). γ-Fe_2_O_3_/II−VI sulfide nanocrystal heterojunctions. J. Am. Chem. Soc..

[16] Moscoso-Londoño O., Ospina C., Brito H.F., Javed Y., Felinto M.C.F.C., Menezes A.S., Knobel M., Sharma S.K. (2017). Building block magneto-luminescent nanomaterials of iron-oxide/ZnS@LaF_3_:Ce^3+^,Gd^3+^,Tb^3+^ with green emission. J. Mater. Chem. C.

[17] An P., Zuo F., Wu Y.P., Zhang J.H., Zheng Z.H., Ding X.B., Peng Y.X. (2012). Fast synthesis of dopamine-coated Fe_3_O_4_ nanoparticles through ligand-exchange method. Chin. Chem. Lett..

[18] Qin Z.L., Du S.N., Luo Y., Liao Z.J., Zuo F., Luo J.B., Liu D. (2016). Hydrothermal synthesis of superparamagnetic and red luminescent bifunctional Fe_3_O_4_@Mn^2+^-doped NaYF_4_:Yb/Er core@shell monodisperse nanoparticles and their subsequent ligand exchange in water. Appl. Surf. Sci..

[19] Wei R., Wei Z., Sun L., Zhang J.Z., Liu J., Ge X., Shi L. (2016). Nile red derivative-modified nanostructure for upconversion luminescence sensing and intracellular detection of Fe^3+^ and MR imaging. ACS Appl. Mater. Interfaces..

[20] Chang H.J., Xie J., Zhao B.Z., Liu B.T., Xu S.L., Ren N., Xie X.J., Huang L., Huang W. (2015). Rare earth ion-doped upconversion nanocrystals: Synthesis and surface modification. Nanomaterials.

[21] Wu Y.Y., Gao D.Y., Zhang P.F., Li C.S., Wan Q., Chen C., Gong P., Gao G.H., Sheng Z.H., Cai L.T. (2016). Iron oxide nanoparticles protected by NIR-active multidentate-polymers as multifunctional nanoprobes for NIRF/PA/MR trimodal imaging. Nanoscale.

[22] Prencipe G., Tabakman S.M., Welsher K., Liu Z., Goodwin A.P., Zhang L., Henry J., Dai H.J. (2009). PEG branched polymer for functionalization of nanomaterials with ultralong blood circulation. J. Am. Chem. Soc..

[23] Ding X.G., Liow C.H., Zhang M.X. (2014). Surface plasmon resonance enhanced light absorption and photothermal therapy in the second near-infrared window. J. Am. Chem. Soc..

[24] Bharathiraja S., Moorthy M.S., Manivasagan P., Seo H., Lee K.D., Oh J. (2017). Chlorin e6 conjugated silica nanoparticles for targeted and effective photodynamic therapy. Photodiagn. Photodyn. Therapy.

[25] Ju Q., Tu D.T., Liu Y.S., Li R.F., Zhu H.M., Chen J.C., Chen Z., Huang M.D., Yu X. (2012). Amine-functionalized lanthanide-doped KGdF_4_ nanocrystals as potential optical/magnetic multimodal bioprobes. J. Am. Chem. Soc..

[26] Yin D.D., Wang C.C., Ouyang J., Song K.L., Liu B., Cao X.Z., Zhang L., Han Y.L., Long X., Wu M.H. (2014). Enhancing upconversion luminescence of NaYF_4_:Yb/Er nanocrystals by Mo^3+^ doping and their application in bioimaging. Dalton Trans..

[27] Luo Y., Du S.N., Zhang W., Liao Z.F., Zuo F., Yang S.T. (2017). Core@shell Fe_3_O_4_@Mn^2+^-doped NaYF_4_:Yb/Tm nanoparticles for triple-modality T_1_/T_2_-weighted MRI and NIR-to-NIR upconversion luminescence imaging agents. RSC Adv..

[28] Shen J., Sun L.D., Zhang Y.W., Yan C.H. (2010). Superparamagnetic and upconversion emitting Fe_3_O_4_/NaYF_4_:Yb,Er hetero-nanoparticles via a crosslinker anchoring strategy. Chem. Commun..

[29] Mi C.C., Zhang J.P., Gao H.Y., Wu X.L., Wang M., Wu Y.F., Di Y.Q., Xu Z.R., Mao C.B., Xu S.K. (2010). Multifunctional nanocomposites of superparamagnetic (Fe_3_O_4_) and NIR-responsive rare earth-doped up-conversion fluorescent (NaYF_4_:Yb,Er) nanoparticles and their applications in biolabeling and fluorescent imaging of cancer cells. Nanoscale.

[30] Serena A., Corr Y.P., Rakovich Y.K., Gun K. (2008). Multifunctional magnetic-fluorescent nanocomposites for biomedical applications. Nanoscale Res. Lett..

[31] Tian G., Gu Z.J., Zhou L.J., Yin W.Y., Liu X.X., Yan L., Jin S., Ren W.L., Xing G.M., Li S.J. (2012). Mn^2+^ dopant-controlled synthesis of NaYF_4_:Yb/Er upconversion nanoparticles for in vivo imaging and drug delivery. Adv. Mater..

[32] Reddy K.L., Rai M., Prabhakar N., Arppe R., Rai S.B., Singh S.K., Rosenholm J.M., Krishnan V. (2016). Controlled synthesis, bioimaging and toxicity assessments in strong red emitting Mn^2+^ doped NaYF_4_:Yb^3+^/Ho^3+^ nanophosphors. RSC Adv..

[33] Hu D., Chen M., Gao Y., Li F.Y., Wu L.M. (2011). A facile method to synthesize superparamagnetic and up-conversion luminescent NaYF_4_:Yb,Er/Tm@SiO_2_@Fe_3_O_4_ nanocomposite particles and their bioapplication. J. Mater. Chem..

[34] Xu X., Lei P.P., Dong L.L., Liu X.L., Su Y., Song S.Y., Feng J., Zhang H.J. (2016). Rational design of Nd^3+^-sensitized multifunctional nanoparticles with highly dominant red emission. Dalton Trans..

[35] He F., Li C.X., Zhang X.Y., Chen Y.Y., Deng X.R., Liu B., Hou Z.Y., Huang S.S., Jin D.Y., Lin J. (2016). Optimization of upconversion luminescence of Nd^3+^-sensitized BaGdF_5_-based nanostructures and their application in dual-modality imaging and drug delivery. Dalton Trans..

[36] Zhou J., Liu Z., Li F.Y. (2012). Upconversion nanophosphors for small-animal imaging. Chem. Soc. Rev..

[37] Xia L., Kong X.G., Liu X.M., Tu L.P., Zhang Y.L., Chang Y.L., Liu K., Shen D.Z., Zhao H.Y., Zhang H. (2014). An upconversion nanoparticle–Zinc phthalocyanine based nanophotosensitizer for photodynamic therapy. Biomaterials.

[38] Joshi P., Ahmadov T.O., Wang P., Zhang P. (2015). Singlet oxygen generation under NIR light and visible light excitations of photosensitizers on upconversion nanoparticle surface. RSC Adv..

[39] Zhao Z., Han Y., Lin C., Hu D., Wang F., Chen X.L., Chen Z., Zheng N.F. (2012). Multifunctional core–shell upconverting nanoparticles for imaging and photodynamic therapy of liver cancer cells. Asian J. Chem..

[40] Collins H.A., Khurana M., Moriyama E.H., Mariampillai A., Dahlstedt E., Balaz M., Kuimova M.K., Drobizhev M., Yang V.X.D., Phillips D. (2008). Blood-vessel closure using photosensitizers engineered for two-photon excitation. Nat. Photonics.

[41] Wang D., Zhu L., Pu Y., Wang J.X., Chen J.F., Dai L.M. (2017). Transferrin-coated magnetic upconversion nanoparticles for efficient photodynamic therapy with near-infrared irradiation and luminescence bioimaging. Nanoscale.

[42] Fowleya C., Nomikoub N., McHalea A.P., McCaughana B., Callan J.F. (2013). Extending the tissue penetration capability of conventional photosensitisers: A carbon quantum dot–protoporphyrin IX conjugate for use in two-photon excited photodynamic therapy. Chem. Commun..

