# Supplementary material for: Role of Mn2+ Doping in the Preparation of Core-Shell Structured Fe3O4@upconversion Nanoparticles and Their Applications in T1/T2-Weighted Magnetic Resonance Imaging, Upconversion Luminescent Imaging and Near-Infrared Activated Photodynamic Therapy
Source: Nanomaterials (Basel). 2018 Jun 26;8(7):466. doi: 10.3390/nano8070466 (PMC6070927; doi:10.3390/nano8070466)
Supplement: Supplementary file 1 [file nanomaterials-08-00466-s001.zip › nanomaterials-314460-supplementary.docx]

Role of Mn^2+^ Doping in the Preparation of Core-Shell Structured Fe_3_O_4_@upconversion Nanoparticles and Their Applications in T_1_/T_2_-Weighted Magnetic Resonance Imaging, Upconversion Luminescent Imaging and Near-Infrared Activated Photodynamic Therapy

Yang Luo ^†^, Wei Zhang ^†^, Zhengfang Liao, Shengnan Yang, Shengtao Yang, Xinhua Li,
Fang Zuo * and Jianbin Luo *

College of Chemistry & Environment Protection Engineering, Southwest Minzu University, Chengdu 610041, China; polymerluoyang@163.com (Y.L.); w070812138@163.com (W.Z.); LiaoZfpolymer@163.com (Z.F.L.); yangsn1993@163.com (S.N.Y.); yangst@pku.edu.cn (S.T.Y.); lxh1905@126.com (X.H.L.)

***** Correspondence: polymerzf@swun.cn (F.Z.); luojb1971@163.com (J.B.L.); Tel.: +86-028-8552-3792 (F.Z.)

† These authors contributed equally to this work.

**Abstract:** Core-shell (C/S) structured upconversion coated Fe_3_O_4_ nanoparticles (NPs) are of great interest due to their potential as magnetic resonance imaging (MRI) and upconversion luminescent (UCL) imaging agents, as well as near-infrared activated photodynamic therapy (PDT) platforms. When C/S structured Fe_3_O_4_@Mn^2+^-doped NaYF_4_:Yb/Er NPs were prepared previously, well-defined C/S-NPs could not be formed without the doping of Mn^2+^ during synthesis. Here, the role of Mn^2+^ doping on the synthesis of core-shell structured magnetic-upconversion nanoparticles (MUCNPs) is investigated in detail. Core-shell-shell nanoparticles (C/S/S-MUCNPs) with Fe_3_O_4_ as the core, an inert layer of Mn^2+^-doped NaYF_4_ and an outer shell consisting of Mn^2+^-doped NaYF_4_:Yb/Er were prepared. To further develop C/S/S-MUCNPs applications in the biological field, amphiphilic poly(maleic anhydride-alt-1-octadecene) (C_18_PMH) modified with amine functionalized methoxy poly(ethylene glycol) (C_18_PMH-mPEG) was used as a capping ligand to modify the surface of C/S/S-MUCNPs to improve biocompatibility. UCL imaging, T_1_-weighted MRI ascribed to the Mn^2+^ ions and T_2_-weighted MRI ascribed to the Fe_3_O_4_ core of C/S/S-MUCNPs were then evaluated. Finally, chlorine e6 (Ce6) was loaded on the C/S/S-MUCNPs and the PDT performance of these NPs was explored. Mn^2+^ doping is an effective method to control the formation of core-shell structured MUCNPs, which would be potential candidate as multifunctional nanoprobes for future T_1_/T_2_-weighted MR/UCL imaging and PDT platforms.

**Keywords:** Mn^2+^; Fe_3_O_4_; upconversion; core-shell-shell; MR/UCL imaging; PDT platforms

**Scheme S1**

**
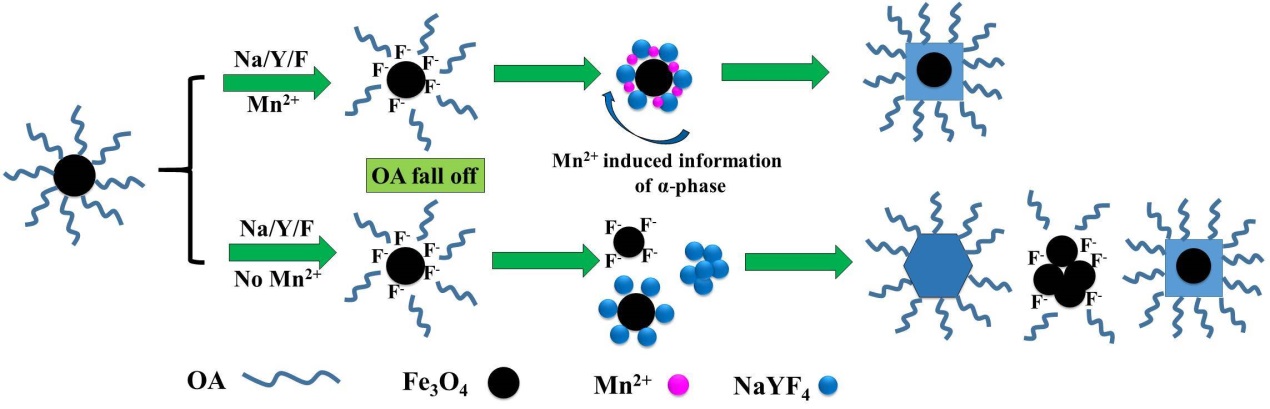
**

**Scheme S1.** Proposed synthetic mechanism for the formation of Fe_3_O_4_@NaYF_4_ NPs.

**Figure S1**

**
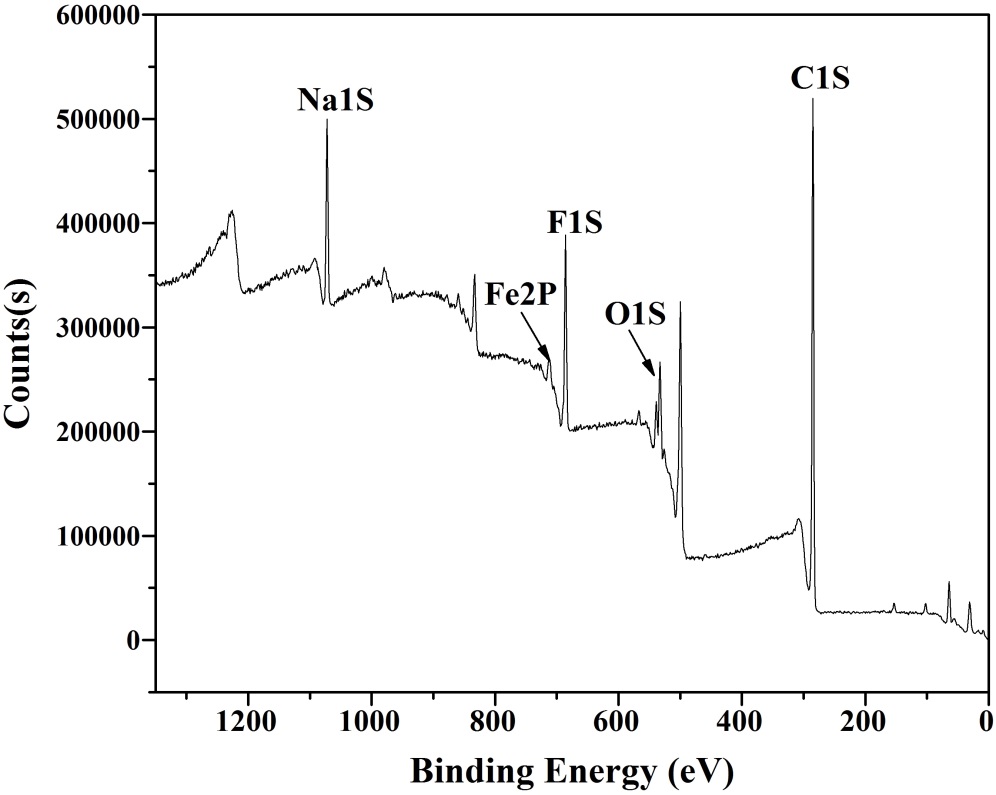
**

**Figure S1.** XPS spectra of Fe_3_O_4_ NPs treated by hydrothermal process in the presence of NaF.

**Figure S2**

**
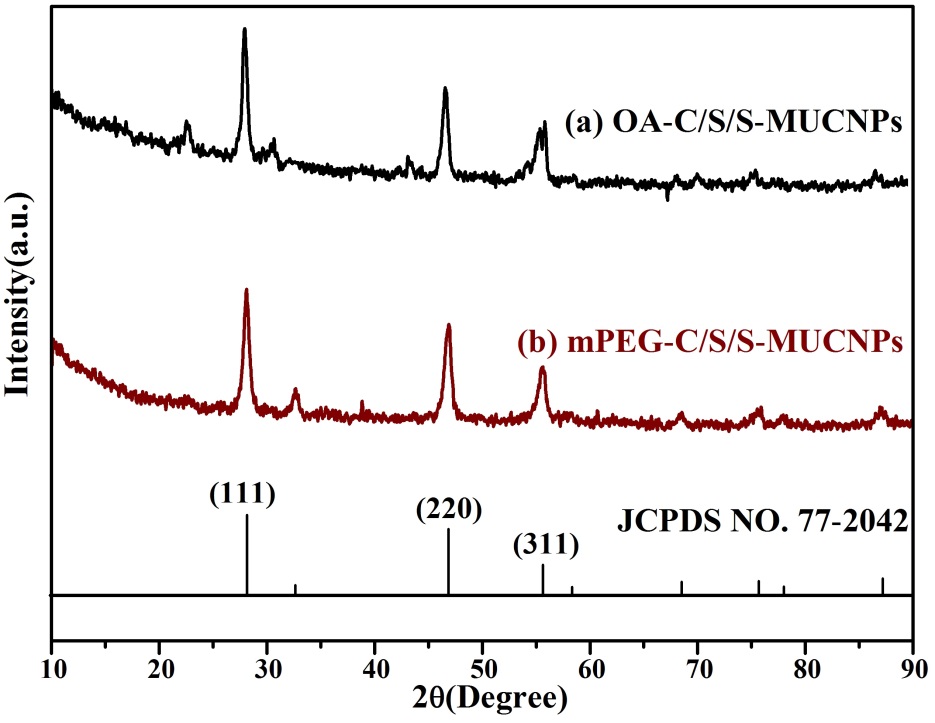
**

**Figure S2.** X-ray diffraction patterns of OA-C/S/S-MUCNPs (**a**) and mPEG-C/S/S-MUCNPs (**b**).

**Figure S3**

**
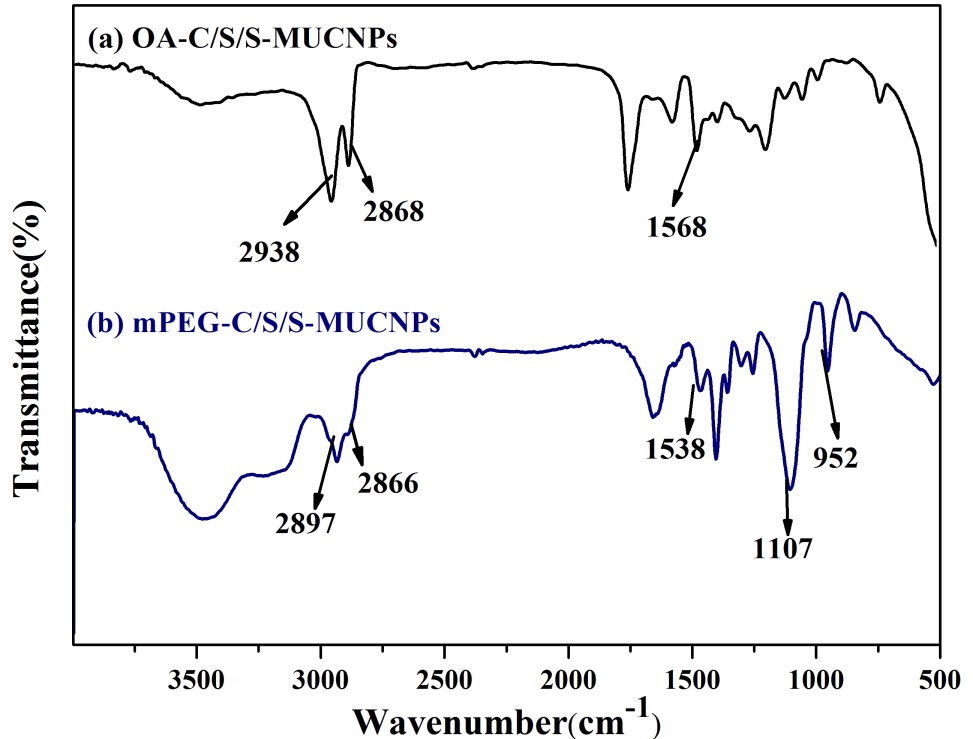
**

**Figure S3.** FTIR spectra of OA-C/S/S-MUCNPs (**a**) and mPEG-C/S/S-MUCNPs (**b**).

**Figure S4**

**
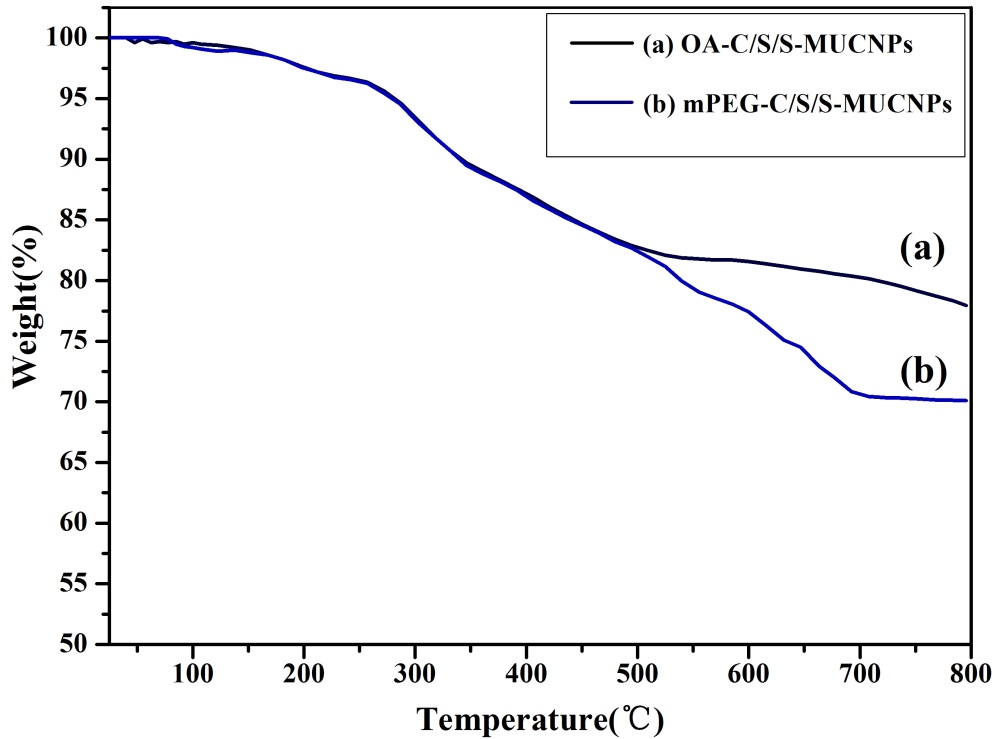
**

**Figure S4.** TGA curves of OA-C/S/S-MUCNPs (**a**) and mPEG-C/S/S-MUCNPs (**b**).

**Figure S5**

**
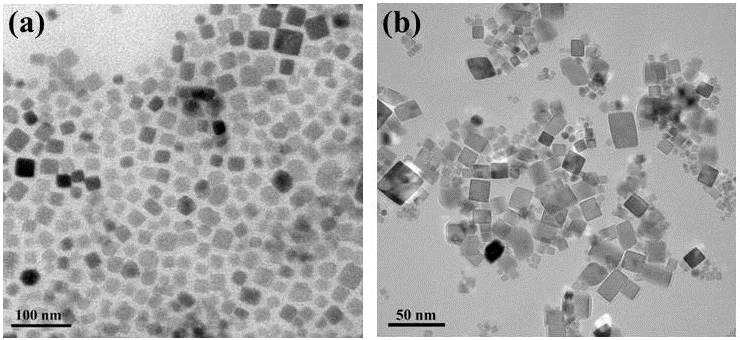
**

**Figure S5.** TEM images of OA-C/S/S-MUCNPs (**a**) and mPEG-C/S/S-MUCNPs (**b**).

**Figure S6**


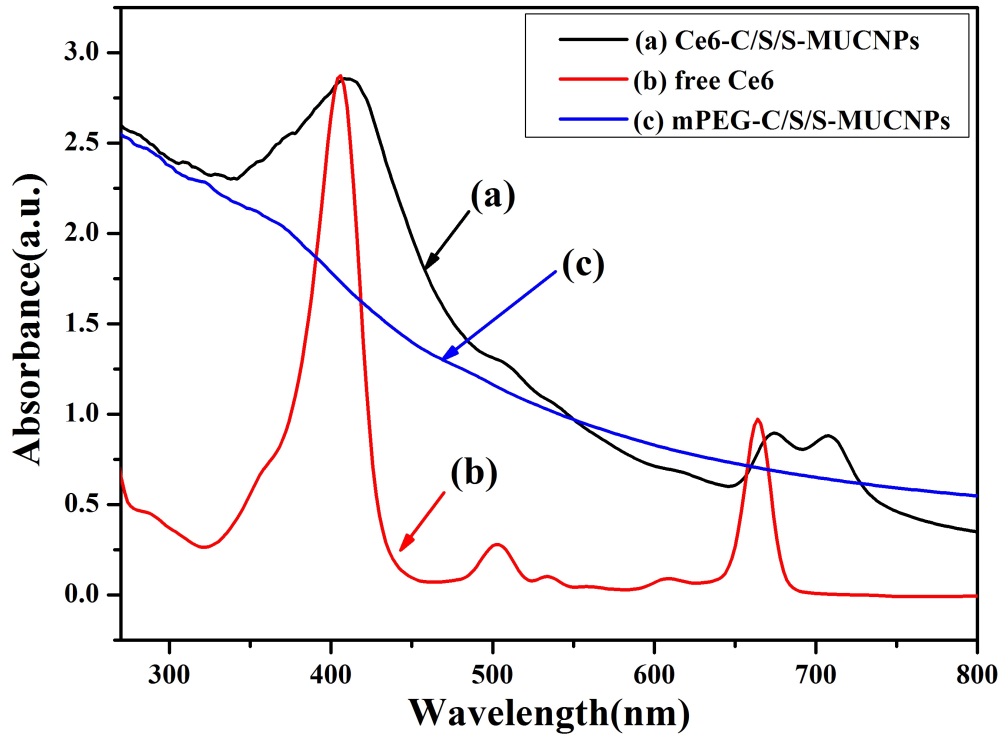


**Figure S6.** UV-Vis absorbance spectrum of Ce6-C/S/S-MUCNPs (**a**), Ce6 (**b**), and mPEG-C/S/S-MUCNPs (**c**).
